# Prospective comparison of liver stiffness measurement methods in surveillance biopsies after liver transplantation

**DOI:** 10.3389/frtra.2023.1148195

**Published:** 2023-11-17

**Authors:** Emily A. Bosselmann, Bastian Engel, Björn Hartleben, Heiner Wedemeyer, Elmar Jaeckel, Benjamin Maasoumy, Andrej Potthoff, Steffen Zender, Richard Taubert

**Affiliations:** ^1^ Department of Gastroenterology, Hepatology, Infectious Diseases and Endocrinology, Hannover Medical School, Hannover, Germany; ^2^ Institute for Pathology, Hannover Medical School, Hannover, Germany

**Keywords:** transient elastography, acoustic radiation force impulse, graft injury, liver cirrhosis, liver fibrosis

## Abstract

**Background:**

Liver stiffness measurements (LSMs) have proven useful for non-invasive detection of fibrosis. Previous studies of LSMs after transplantation were performed in cohorts dominated by hepatitis C reinfections and indication biopsies for the evaluation of graft dysfunction. However, the diagnostic fidelity of LSMs for fibrosis is biased by inflammation e.g., during replicative hepatitis C or rejection.

**Materials and methods:**

The current study aimed for a head-to-head comparison of two different LSMs, acoustic radiation force impulse (ARFI) and transient elastography (TE), and a determination of cut-off values for the detection of advanced fibrosis (any LAF score component ≥2) in grafts undergoing surveillance biopsies (svLbx) without recurrent hepatitis C.

**Results:**

103 svLbx were paired with valid LSMs at time of biopsy. AUROC analyses showed significant positive correlation with fibrosis for both methods (TE: AUROC = 0.819 (*p* < 0.001; 95%CI: 0.717–0.921); ARFI: AUROC = 0.771 (*p* = 0.001; 95%CI: 0.652–0.890). Patients were randomly assigned to training and validation cohorts for both LSM methods. Cut-off values were determined at 1.29 m/s (ARFI) and at 7.5 kPa (TE) in training cohorts. Sensitivity and specificity in training and validation cohorts were: TE: SEN 0.818 and 0.5; SPE 0.742 and 0.885; ARFI: SEN 0.818 and 1.0; SPE 0.75 and 0.586. LSMs were not associated with BANFF criteria for relevant graft injury.

**Conclusion:**

LSM is a good non-invasive tool to screen for advanced graft fibrosis but not for relevant graft injury in patients with (near) normal liver enzymes. Fibrosis cut-off values identified and validated in svLbx were lower than in previous cohorts using indication biopsies.

## Introduction

Although most complications after orthotopic liver transplantation (OLT), such as infections, kidney failure, and malignancy, are associated with immunosuppressive therapy, graft fibrosis remains a major cause of re-transplantation or death after liver transplantation (1). Graft biopsy is the gold standard for the detection of significant fibrosis and cirrhosis. However, liver biopsies (Lbx) are expensive and carry the risk of complications, such as bleeding or pain, even though these risks have proven to be very low (2, 3). For many years, liver stiffness measurements (LSMs) have been used as practical non-invasive surrogate parameters for liver fibrosis.

Transient elastography (TE) is a well-known method for detecting liver fibrosis in patients with different underlying diseases and even after OLT. In addition, it allows the examination of a large volume of liver tissue. However, TE measurements can fail due to a high body mass index, ascites, or narrow intercostal spaces (2). Previous studies have also described correlations to bile tract anomalies and acute hepatitis (3).

Acoustic radiation force impulse (ARFI) has also been evaluated for the detection of liver fibrosis in several different studies (4). It is ultrasound-based and can therefore be combined with regular ultrasonographic examinations before and after liver transplantation. The ultrasound probe generates high-energy acoustic pulses which trigger localised tissue displacements. Through this, shear waves are propagated and their velocity can be measured in a certain region of interest. Results are described in m/s (5).

Several studies have proven a correlation between liver stiffness measurements and fibrosis before and after OLT. However, these studies were performed in cohorts predominantly including patients with chronic hepatitis C before the introduction of direct-acting antivirals (DAA) and mostly in comparison to indication biopsies (4, 6). The achievement of sustained virologic response in hepatitis C is, however, associated with an improvement of LSM (7).

The aim of this study was to prospectively compare TE and ARFI by including these examinations in our surveillance biopsy (svLbx) program after OLT in the post-DAA era.

## Material and methods

### Subjects

We included all adult liver recipients without a replicative viral hepatitis (HCV-RNA or HBs-Ag negativity) who underwent at least one surveillance liver biopsy and agreed to participate in our prospective liver allograft biorepository data-base and in this prospective observational study from November 2018 to September 2020 (age at time of liver biopsy ≥18 years). A participation in the surveillance biopsy (svLbx) program was voluntary and offered to all liver transplanted patients without contraindications, e.g., severely dilated bile ducts, thrombocytopenia etc. For scientific analysis indication and surveillance biopsies were distinguished based on liver enzyme levels in paired blood samples. While AST and ALT were normal or near normal [<2-fold upper limit of normal (ULN)] and AP and GGT were at stable levels (even though elevated in some patients), indication biopsies had liver enzymes above this threshold. Similar thresholds were used in studies on intentional immunosuppression (IS) withdrawal and in previous analyses of our program (3, 8, 9). Our svLbx program was established in 2018 and aims to perform svLbx in patients at specific time points after OLT, i.e., one, three, five and ten years post-OLT and then every 5 years. All biopsies are then discussed in monthly interdisciplinary conferences and IS is adapted accordingly. Patients without graft injury are advised to reduce IS in order to minimize adverse effects of the medication. Patients with significant graft fibrosis are mostly advised to switch their IS regimen to a combination of a calcineurin inhibitor (CNI) and an m-TOR inhibitor (i.e., everolimus) due to the antifibrotic effects of m-TOR inhibitors (9–12). Patients with subclinical graft injury are mainly advised to stay on their current IS regimens, unless there are important reasons to change or reduce IS, i.e., malignant diseases or major infections (9).

This study was approved by the Ethics Committee of our center. Written informed consent was obtained from all subjects. All experiments were performed in accordance with relevant guidelines and regulations. No organs or tissues were procured from prisoners.

### Liver stiffness measurements

After an overnight fast, the patients were laid in a supine position with their arms maximally abducted under their head. The success rate was calculated as the number of successful measurements divided by the total number of measurements. Only procedures with at least 10 valid acquisitions, a success rate of greater than 60% and an interquartile range (IQR/M) less than 30% were defined as eligible for the study.

According to study protocol, each patient was to receive both their ARFI and TE measurements from different examiners within 7 days prior to liver biopsy.

ARFI elastography was performed with Virtual Touch tissue quantification (Siemens Acuson S2000, Siemens Healthineers, Erlangen, Germany) with a standard broad-band 4–1 MHz curved array, as described recently (13). This allowed placement of the region of interest under vision in ultrasonographic B-mode. Measurements were taken in liver segment 8 through an intercostal approach without a specific breathing maneuver. Care was taken to minimize the pressure exerted during measurement and to avoid region of interest placement on large vessels, biliary radicles, and focal lesions (if present). The results were given in meters per second (m/s).

Transient elastography (TE) was measured using Fibroscan® 502 Touch (Echosens, France) with a low-frequency vibrator (50 Hz) for the excitation of shear waves and an ultrasonic single-element transducer operating at 5 MHz on the axis of the vibrator as described recently (13). The results were given in kilopascals (kPa).

### Liver biopsy specimens

Liver biopsies were performed percutaneously and ultrasound-guided under local anesthesia with a 16-gauge needle, fixed in 4% neutral buffered formalin and embedded in paraffin wax.

### Histological grading and staging

Histological grading and staging was performed as described recently (14). Sections of 2 µm thickness from liver allograft biopsies were stained with hematoxylin and eosin, elastic van Gieson stain, periodic acid–Schiff stain, silver stain, Berlin blue stain and rhodamine stain. Histological examination was performed by experienced liver pathologists in a blinded fashion. Only Lbx regarded as representative by the examining pathologist, including at least 5 portal fields, were included. The liver tissue was examined according to the Ishak scoring system, as well as liver allograft fibrosis score (LAFSc) and Banff schema for grading liver allograft rejection with the rejection activity index (RAI) (15). The RAI score was constituted by examining portal, bile duct and venous endothelial inflammation with a maximum of 3 points, respectively (16). Patients with at least one point in each of the three categories, therefore showing morphological signs of graft rejection, and non-elevated liver enzymes (AST and ALT≤2x ULN) were diagnosed with subclinical T cell-mediated rejection, as described in previous studies (17). LAFSc was scored by separately assessing portal, sinusoidal and centrilobular areas, each with a maximum of 3 points, allowing the maximum score to lie at 9 points for both RAI and LAFSc (15). For this study, significant fibrosis was defined as any LAFSc component ≥2. The much used Ishak scoring system characterizes fibrosis on a scale from 0 to 6 (18). For this study, at least moderate fibrosis was defined as periportal fibrosis (Ishak *F*) ≥ 2. Significant fatty degeneration of graft tissue was diagnosed at an augmentation of lipid vacuoles in hepatocytes of >5% (19). Any biopsy not fulfilling the criteria justifying the minimization of immunosuppression (“BanffMini”: portal tract inflammation ≤ 1, interface hepatitis ≤ 1, central perivenulitis ≤ 1, lobular inflammation = 0, biliary inflammation = 0, endothelialitis = 0, portal microvasculitis = 0 and periportal fibrosis ≤ 3) was diagnosed with significant subclinical graft injury (16).

### Statistical analysis

Statistical analysis was performed using SPSS 15.0. The Mann–Whitney *U* test was used to compare quantitative data between two groups. The Chi^2^ test was used to prepare contingency tables with two groups. Correlation analyses were calculated with Spearman's rank correlation. Area under the receiver operating characteristic (AUROC) analyses and the Youden's index were used to guide identification of cut-off values. *P*-values below 0.05 (two-tailed) were considered significant in all analyses.

## Results

**Figure 1:**
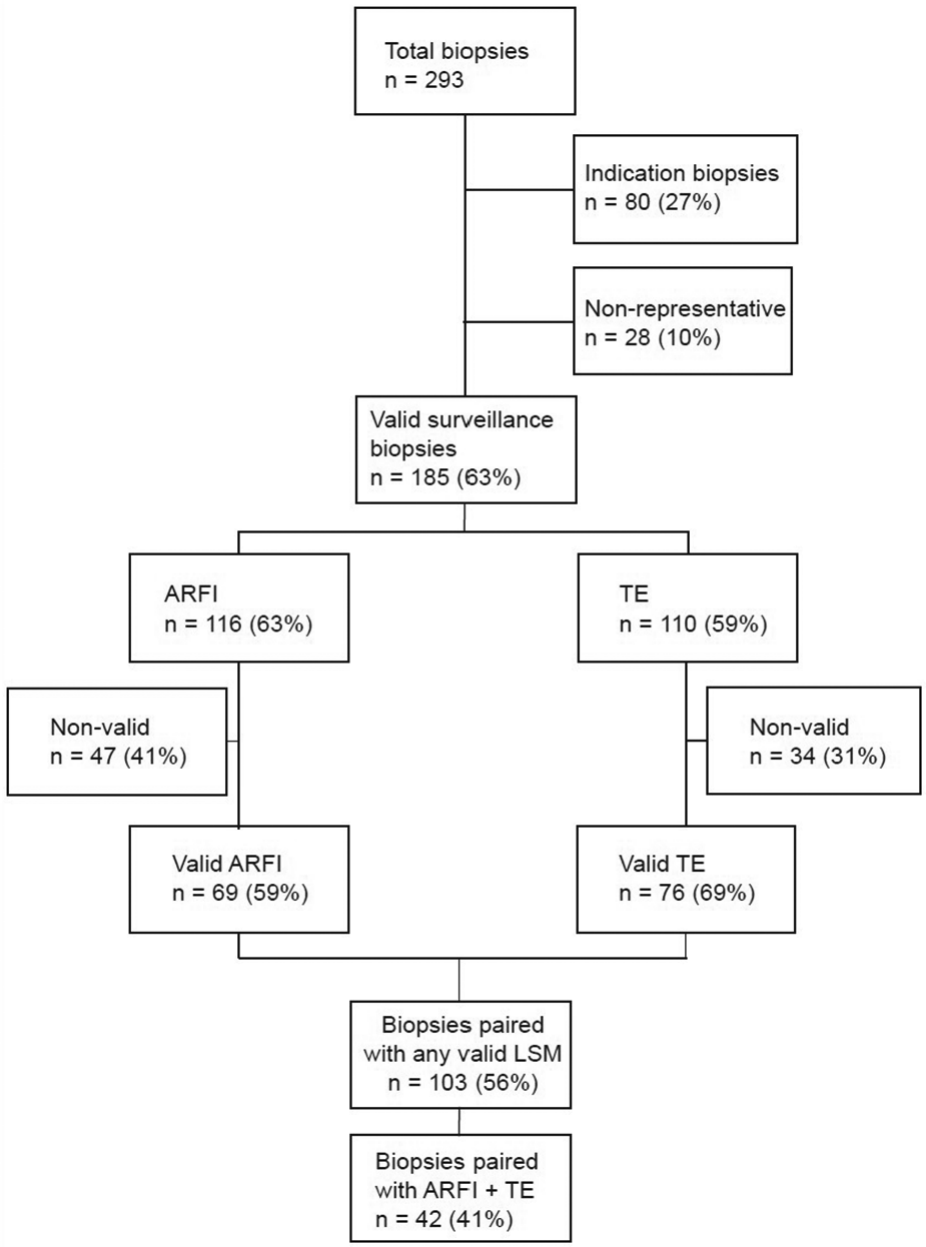
Flow chart outlining availability and selection of patients.

From 11/2018 to 09/2020, 293 Lbx were performed on 287 patients at our center. All biopsies regarded in this study were surveillance Lbx (svLbx), performed to adjust the immunosuppression regimen without relevantly elevated liver enzymes or previously elevated LSMs (Table 1). Twenty-eight (10%) Lbx were excluded from this study due to non-representative graft biopsy. In total, 226 LSMs were carried out. Baseline patient characteristics are shown in Table 1. Valid LSMs were performed within 4 days prior to Lbx (median: 0; range: 0–4). Overall, one hundred three surveillance biopsies could be paired with at least one valid LSM (ARFI and/or TE). In 42 (41%) cases, both ARFI and TE were matched (Figure 1).

**Table 1:** Baseline characteristics of patients included in the study (*n* = 103).

** **	ARFI (*n* = 69)	TE (*n* = 76)	*p*-values ARFI vs. TE
Age at biopsy (years)	54 (18–72)	52 (18–72)	0.337*
18–30 years *n* (%)	6 (8.7)	9 (11.8)	0.534
31–50 years *n* (%)	20 (29)	23 (30.3)	0.866
51–72 years *n* (%)	43 (62.3)	44 (57.9)	0.587
Male gender *n* (%)	43 (62.3)	45 (59.2)	0.702
BMI at biopsy (kg/m^2^)	23.8 (18.6–33.3)	24.5 (18.4–34.4)	0.689*
Underlying disease *n* (%)			
Autoimmune liver disease	25 (36.2)	22 (28.9)	0.349
Chronic viral hepatitis	17 (24.6)	15 (19.7)	0.477
Cryptogenic	9 (13)	8 (10.5)	0.638
Alcoholic liver disease	4 (5.8)	3 (3.9)	0.604
Non-alcoholic fatty liver disease	2 (2.9)	3 (3.9)	0.730
Cystic liver disease	3 (4.3)	4 (5.3)	0.797
(Congenital) metabolic disorders	4 (5.8)	10 (13.2)	0.134
Biliary cirrhosis	3 (4.3)	3 (3.9)	0.904
Toxic liver disease	1 (1.4)	3 (3.9)	0.359
Other	1 (1.4)	4 (5.3)	0.209
Age at OLT (years)	48 (0–67)	44 (0–67)	0.441*
0–17 years *n* (%)	6 (8.7)	8 (10.5)	0.709
18–30 years *n* (%)	9 (13)	11 (14.5)	0.803
31–50 years *n* (%)	28 (40.6)	32 (42.1)	0.852
51–67 years *n* (%)	26 (37.7)	25 (32.9)	0.547
Graft type *n* (%)			
Whole graft	59 (85.5)	68 (89.5)	0.469
Right split	9 (13)	6 (7.9)	0.309
Left split	1 (1.4)	2 (2.6)	0.617
Time from OLT to biopsy (months)	103 (11–452)	82 (9–406)	0.741*
0–24 months *n* (%)	14 (20.3)	14 (18.4)	0.776
25–60 months *n* (%)	12 (17.4)	18 (23.7)	0.350
61–120 months *n* (%)	13 (18.8)	15 (19.7)	0.891
> 120 months *n* (%)	30 (43.5)	29 (38.2)	0.515
AST (U/L)	24 (12–55)	24 (8–60)	0.610*
ALT (U/L)	21 (12–72)	21 (8–72)	0.689*
AP (U/L)	81 (32–473)	91 (46–263)	0.289*
GGT (U/L)	22 (7–377)	23 (7–927)	0.920*
Bilirubin (µmol/L)	8 (3–55)	9 (3–55)	0.865*

Values are described as median (range), unless indicated differently.

*p*-values were calculated by Chi^2^ test, except*.

* *p*-values calculated by Mann–Whitney-*U* test.

In the overall cohort (*n* = 103), main reasons for transplantation were autoimmune liver diseases (*n* = 27; 26%), viral hepatitis (*n* = 22; 21%), other cirrhosis (*n* = 17; 17%) and hepatocellular carcinoma (*n* = 12; 12%).

Out of 110 TEs performed, 34 (31%) measurements were deemed non-valid: 16 (47%) for higher time lag between LSM and biopsy than intended, one (3%) for non-fasting patient, 9 (26%) for low number of measurements and 8 (23%) for high IQR-to-median ratio. Results of valid TE showed a median of 6.1 kPa (range: 3.4–26.9 kPa). Out of 116 ARFI measurements carried out, 47 (41%) were non-valid: 4 (9%) for higher time lag between LSM and biopsy than intended, 3 (6%) for low number of measurements and 40 (85%) for high IQR-to-median ratio. Median ARFI values were at 1.25 m/s (range: 0.67–4.07 m/s). Out of 34 invalid TEs, 24 (70%) underwent ARFI examination, leading to valid measurements in 13 (54%) cases. Vice versa, in the cohort of invalid ARFI (*n* = 47), 31 (66%) underwent TE with 20 (65%) valid measurements.

Histological assessment showed graft fibrosis (Ishak *F* 2–4) in 19 (18%) svLbx and cirrhosis (Ishak *F* ≥ 5) in 3 (3%) svLbx (Table 2). The liver allograft fibrosis score (LAFSc) showed a median of 1 (range: 0–6) point (15). Twenty-seven (26%) patients showed advanced graft fibrosis, defined as any LAFSc component ≥2. Any kind of relevant subclinical graft injury according to the 2016 Banff consensus (beyond BanffMini) was detected in 65 (63%) cases (Table 2) (16).

**Table 2:** Histological findings.

** **	ARFI (*n* = 69)	TE (*n* = 76)	Total cohort (*n* = 103)
Fibrosis (Ishak *F*2–4) *n* (%)	9 (13)	16 (21.1)	19 (18.4)
Fibrosis (any LAFSc component ≥2) *n* (%)	15 (22)	19 (25)	28 (27)
Cirrhosis (Ishak *F*5–6) *n* (%)	3 (4.3)	1 (1.3)	3 (2.9)
Beyond BanffMini *n* (%)	44 (63.8)	50 (65.8)	65 (63.1)
Disease recurrence (suspected) *n* (%)	2 (2.9)	2 (2.6)	3 (2.9)

Percentages are shown in brackets.

**Figure 2:**
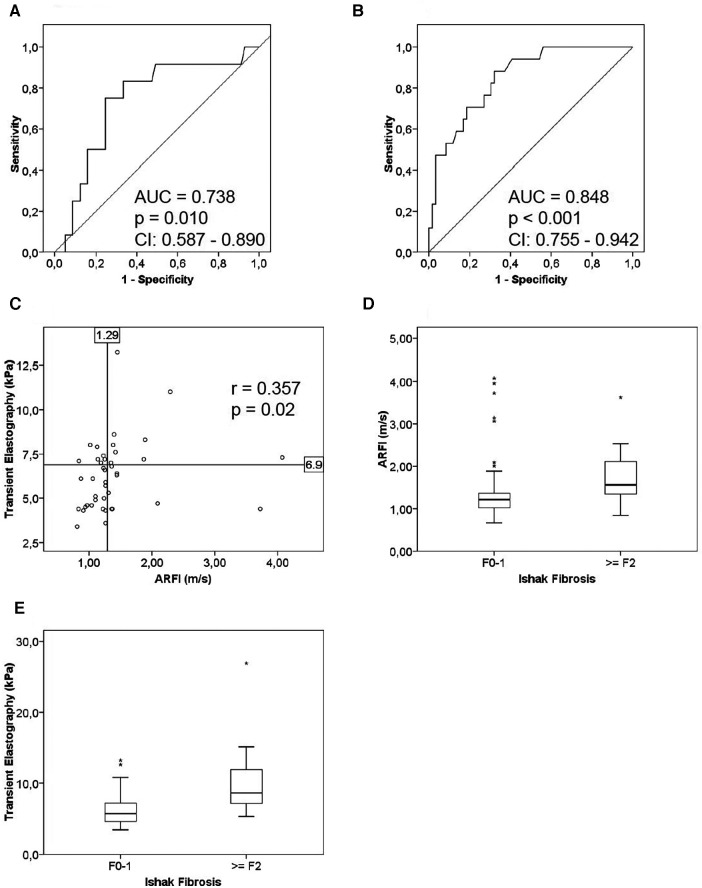
ROC curves and correlation analysis for liver stiffness measurements. (**A**) ARFI: the ROC curve was calculated for valid ARFI measurements (*n* = 69) and histological grading of fibrosis (Ishak *F*). Fibrosis was considered relevant at Ishak *F*2 or higher. Histopathological evaluation yielded Ishak *F* ≤ 1 for 57 (83%) and Ishak *F* ≥ 2 for 12 (17%) patients. (**B**) TE: the ROC curve was calculated for valid TE measurements (*n* = 76) and histological grading of fibrosis (Ishak *F*). Fibrosis was considered relevant at Ishak *F*2 or higher. Histopathological evaluation yielded Ishak *F* ≤ 1 for 59 (78%) and Ishak *F* ≥ 2 for 17 (22%) patients. (**C**) ARFI and TE: the scatter plot shows correlation between ARFI and TE (*n* = 42), calculated using Spearman's correlation analysis. (**D**) Boxplot showing the range of ARFI measurements for Ishak *F*0–1 (*n* = 57) and Ishak *F* ≥ 2 (*n* = 12). (**E**) Boxplot showing the range of TE measurements for Ishak *F*0–1 (*n* = 59) and Ishak *F* ≥ 2 (*n* = 17). (CI = 95% confidence interval).

**Figure 3:**
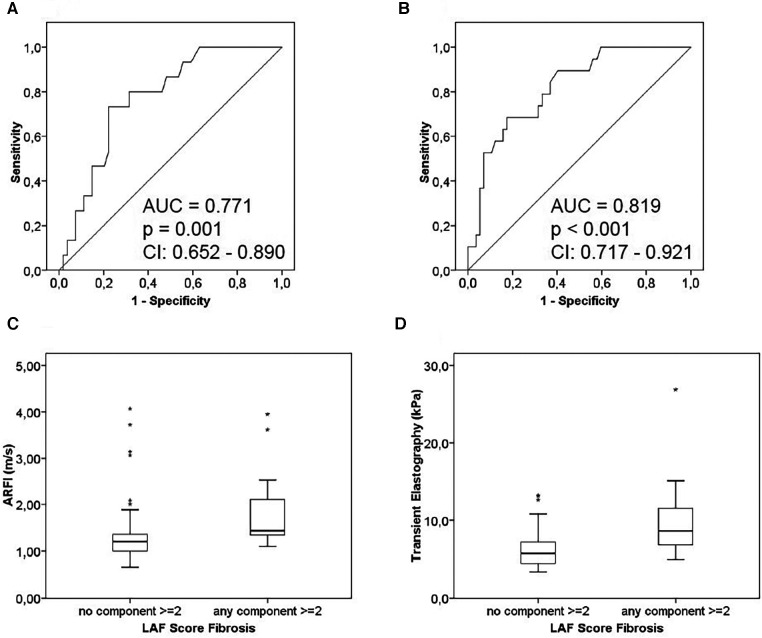
ROC curves showing correlation of liver stiffness measurements with graft fibrosis (LAF score). (**A**) ARFI: the ROC curve was calculated for valid ARFI measurements (*n* = 69) and significant fibrosis according to LAFSc. Histopathological evaluation yielded significant fibrosis (any LAFSc component ≥2) for 15 (22%) patients. (**B**) TE: the ROC curve was calculated for valid TE measurements (*n* = 76) and advanced fibrosis according to LAFSc. Histopathological evaluation yielded significant portal fibrosis (any LAFSc component ≥2) for 19 (25%) patients. (**C**) Boxplot showing the range of ARFI measurements for no significant LAFSc fibrosis (*n* = 54) and advanced fibrosis (any LAFSc component ≥2) (*n* = 15). (**D**) Boxplot showing the range of TE measurements for no significant LAFSc fibrosis (*n* = 59) and advanced fibrosis (any LAFSc component ≥2) (*n* = 17). (CI = 95% confidence interval).

AUROC for detection of relevant graft fibrosis according to the Ishak scoring system (Ishak *F* ≥ 2) was 0.738 (*p* = 0.010; 95% CI: 0.578–0.890) for ARFI (*n* = 69) (Figure 2A), while AUROC was 0.848 (*p* < 0.001; 95% CI: 0.755–0.942) (Figure 2B) for TE (*n* = 76). AUROC between ARFI and any LAFSc component ≥2 was 0.771 (*p* = 0.001; 95% CI: 0.652–0.890) (Figure 3A). AUROC for detection of relevant graft fibrosis according to the LAFSc also showed correlation between TE and fibrosis (any LAFSc component ≥2; AUROC = 0.819; *p* < 0.001; 95% CI: 0.717–0.921) (Figure 3B).

Correlation analysis using the Spearman–Rho test showed significant correlation between the two LSM methods (*r* = 0.357, *p* = 0.02) (Figure 2C) and between each LSM and histological graft fibrosis. TE results were correlated with elevation of cholestatic enzymes (Spearman's correlation analysis: AP: *r* = 0.275, *p* = 0.016; GGT: *r* = 0.375, *p* = 0.001), but not bilirubin (*r* = 0.089, *p* = 0.445). ARFI however did not correlate with elevation of cholestatic enzymes (Spearman's correlation analysis: AP: *r* = 0.151, *p* = 0.216; GGT: *r* = 0.199, *p* = 0.102; bilirubin: *r* = 0.164, *p* = 0.179).

To determine and validate a cut-off value for each LSM, the cohorts for ARFI and TE were divided into a training and a validation group respectively for each LSM methodology. Assignment to either training or validation cohort was performed randomly, aiming for 50%–66% of patients to be in the respective training cohorts. Clinical characteristics of training and validation cohorts are outlined in Supplementary Table S1.

For ARFI, the ideal cut-off value for relevant graft fibrosis (any LAFSc component ≥2), was determined at 1.29 m/s using the Youden's index in the ARFI training cohort. Test characteristics describing the predictive fidelity of ARFI with this cut-off value in the training and validation cohort are outlined in Table 3. With the same approach, the ideal cut-off value for any LAFSc component ≥2 was determined at 7.5 kPa in the TE training cohort. Test characteristics of when this cut-off value was applied are also outlined in Table 3.

**Table 3:** Predictive accuracy of cut-off values for ishak *F* ≥ 2.

** **	ARFI (*n* = 69) Cut-off: 1.29 m/s		TE (*n* = 76) Cut-off: 6.9 kPa	
	Training (*n* = 39)	Validation (*n* = 30)	Training (*n* = 42)	Validation (*n* = 34)
AUROC	0.807	0.655	0.856	0.857
Sensitivity	0.818	1.0	0.818	0.833
Specificity	0.75	0.586	0.645	0.75
Positive predictive value	0.563	0.077	0.45	0.417
Negative predictive value	0.913	1.0	0.909	0.955

In 37 patients an ARFI measurement was also available for the left liver lobe, however only 21 (57%) had valid results. The main reason for the measurements not to be valid was IQR-to-median ratio of >30% (*n* = 13; 81%). Due to the small number, the ROC curve did not qualify for safe results (AUROC = 0.740; *p* = 0.080; 95% CI: 0.529–0.951). Cross-tabulations using the cut-off value of right-sided ARFI (1.29 m/s) showed the following results: sensitivity = 1.0; specificity = 0.438; PPV = 0.357; NPV = 1.0.

When these validated LSM cut-offs were applied, bile duct abnormalities described in ultrasound examinations (Chi^2^ test: *p* = 0.749) and fatty degeneration described by the examining pathologist (Chi^2^ test: *p* = 0.072) were not significant confounders of ARFI. However, bile duct abnormalities showed to be a significant confounder of TE (Chi^2^ test: *p* = 0.046), while fatty degeneration did not affect TE results (Chi^2^ test: *p* = 0.448). The prevalence of diabetes (*n* = 12; 12%) however showed to be a confounder of both TE and ARFI: though their LSM values were not significantly higher than those of the overall cohort (TE: 4.6 kPA (range: 4.1–8.0 kPa; *n* = 9) vs. 6.1 kPa (range: 3.4–26.9 kPa; *n* = 76); ARFI: 1.26 m/s (range: 0.97–3.95 m/s; *n* = 8) vs. 1.25 m/s (range: 0.67–4.07 m/s; *n* = 69)), both LSM lost significant correlation with graft fibrosis in this sub-analysis. Additionally, LSM tended to be false-positive when applying the previously mentioned cut-off values.

**Figure 4:**
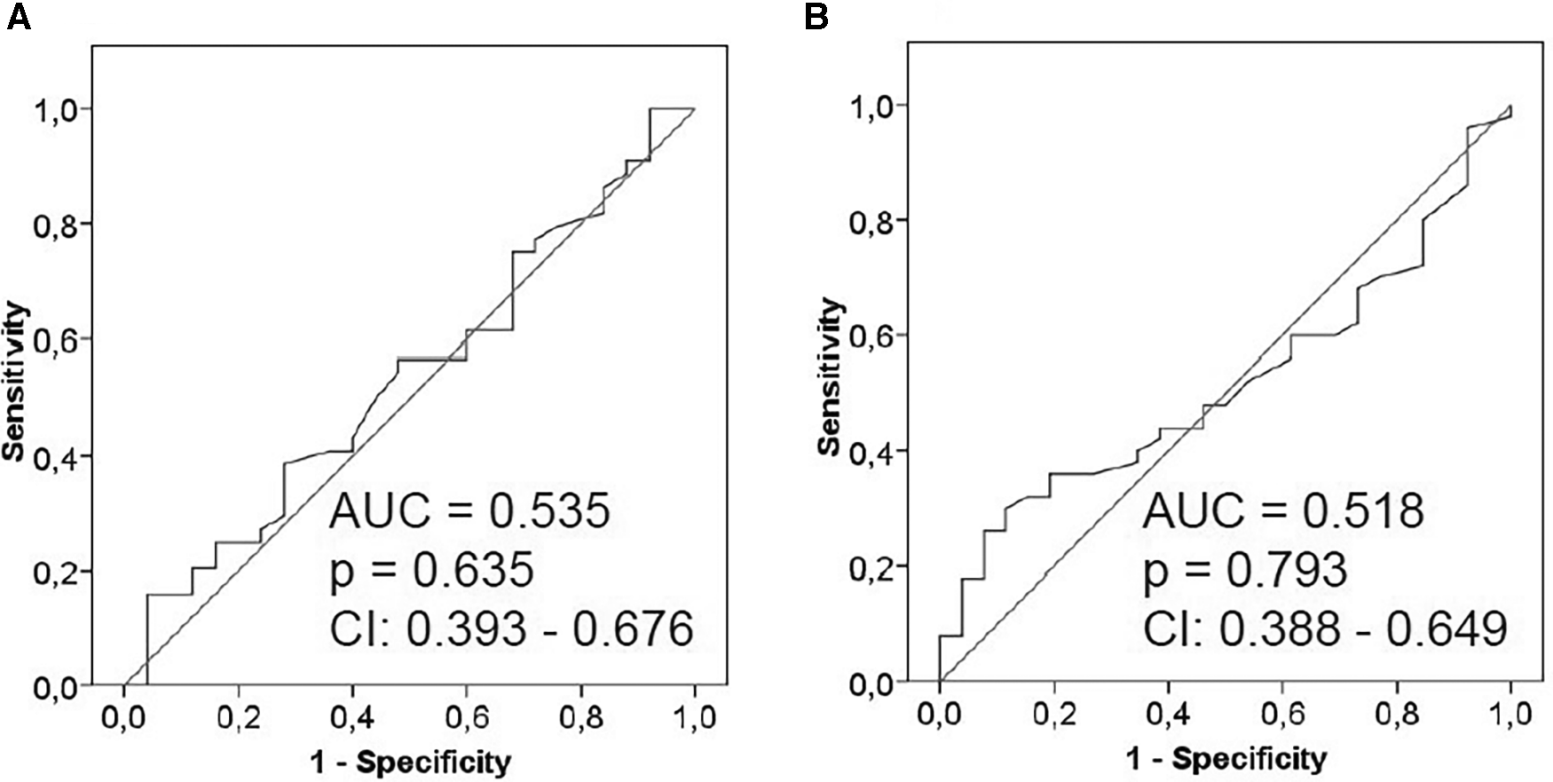
ROC curves showing correlation of liver stiffness measurements with any kind of histological graft injury (beyond banffMini). (**A**) ARFI: the ROC curve was calculated for valid ARFI measurements (*n* = 69) and any kind of histological graft injury (anything beyond BanffMini). Histopathological evaluation yielded BanffMini criteria fulfilled for 25 (36%) and not fulfilled for 44 (64%) patients. (CI = 95% confidence interval) (**B**) TE: the ROC curve was calculated for valid TE measurements (*n* = 76) and any kind of histological graft injury (anything beyond BanffMini). Histopathological evaluation yielded BanffMini criteria fulfilled for 26 (34%) and not fulfilled for 50 (66%) patients. (CI = 95% confidence interval).

To evaluate whether LSM were explicitly applicable only for graft fibrosis, the BanffMini criteria were evaluated for each biopsy. ROC curves showed no significant correlation of either LSM with biopsies beyond BanffMini (ARFI: AUC = 0.535, *p* = 0.635, CI: 0.393–0.676; TE: AUC = 0.518, *p* = 0.793, CI: 0.388–0.649; Figure 4).

## Discussion

This is the first study to compare two common LSMs in a svLbx cohort and to generate and validate cut-off values in separate patient cohorts. In the current study, TE showed a more stringent correlation with graft fibrosis, but looked to be more strongly influenced by cholestasis. The validated cut-off values for the prediction of relevant liver graft fibrosis (Ishak *F* ≥ 2) were 1.29 m/s in ARFI and 6.9 kPa in TE and lower than in a previous study that was still dominated by HCV reinfections (cut-off values: 1.39 m/s (ARFI) and 8.4 kPa (TE)) (20). Cut-off values for advanced fibrosis were 1.29 m/s in ARFI and 7.5 kPa in TE when liver graft fibrosis was quantified according to LAFSc, which is more accurate for fibrosis quantification than the Ishak fibrosis score (15).

Other studies also determined cut-off values for each histological fibrosis stage even after transplantation, but these studies mostly did not validate these detailed cut-off values. We, however, preferred to use the sample size to validate the cut-offs predicting relevant graft fibrosis, rather than to generate several cut-offs. Furthermore, other studies rather included indication biopsies, mostly looking for signs of acute graft rejection, while our svLbx and LSMs were performed in patients with normal/near normal liver values.

Compared to other studies, the sample size for ARFI (*n* = 69) was quite large (Abdelhaleem et al. 2018: *n* = 70; Liao et al. 2014: *n* = 57, Schmillevitch et al. 2016: *n* = 33) (4, 5, 21, 22). The cut-off at 1.29 m/s was more within the lower range of most other studies on ARFI in OLT recipients dominated by HCV reinfection, in which cut-off values ranged between 1.29 m/s and 1.75 m/s (4, 23–25). With regard to TE, some studies have analyzed the correlation with fibrosis after OLT with larger patient numbers, all of them, however, focused on patients with HCV reinfection (75%–100%) (26–28). If we had not excluded the indication biopsies in the present study, the optimal cut-off values for relevant liver graft fibrosis would have been higher for ARFI (1.37 m/s) and similar (6.9 kPa) for TE. [Data previously published elsewhere]

Compared to other studies, the cut-off value established for TE in this study was relatively low, since other results have ranged from 4.7 to 12.3 kPa (20, 29–31). In this study, the cut-off values at 1.29 m/s and 7.5 kPa for advanced fibrosis according to LAFSc (and 1.29 m/s and 6.9 kPa for advanced fibrosis according to Ishak score) showed excellent negative predictive values, so that ruling out significant graft fibrosis using LSMs can be deemed considerably safe (Tables 3, 4). While sensitivity was a little lower, the detection of graft injury is more important for OLT recipients than confirmation of health in graft tissue.

**Table 4:** Predictive accuracy of cut-off values for any LAFSc component ≥2).

** **	ARFI (*n* = 69) Cut-off: 1.29 m/s		TE (*n* = 76) Cut-off: 7.5 kPa	
	Training (*n* = 39)	Validation (*n* = 30)	Training (*n* = 42)	Validation (*n* = 34)
AUROC	0.894	0.525	0.850	0.757
Sensitivity	0.833	0.667	0.833	0.429
Specificity	0.778	0.593	0.767	0.889
Positive predictive value	0.625	0.154	0.588	0.5
Negative predictive value	0.913	0.941	0.92	0.857

Regular testing of liver enzymes paired with TE and/or ARFI can be helpful in between biopsies and in patients declining offers of svLbx. According to our results, since TE was more stringently associated with graft fibrosis, we recommend primarily performing TE. However, if TE does not deliver valid results or if TE is not available at the transplant center, ARFI should be considered. After invalid results in one LSM our results showed valid results in 54%–65% of measurements with the alternative LSM. Furthermore, ARFI can also be used in patients with left-lobe split grafts, although these results should be interpreted with caution. Additionally, LSMs should be complemented by ultrasound analysis to exclude potential confounders of elevated liver stiffness, such as obstructive cholestasis or congestive heart failure, seeing as in this study bile duct modifications looked to confound TE (but not ARFI) results. Especially patients with diabetes should undergo ultrasound examinations in case of increased LSM values, since this study showed diabetic patients to be prone to false-high LSM results, matching a previous meta-analysis on this matter (32).

This study showed significant correlation between LSMs and graft fibrosis. This non-invasive prediction can help to screen liver graft recipients in order to explore the causes of liver graft fibrosis such as insufficiently controlled alloreactivity or disease recurrence (17, 33–35). When LSM is above the defined cut-off value (1.29 m/s and 7.5 kPa respectively; the respective cut-offs for advanced fibrosis according to Ishak score were 1.29 m/s and 6.9 kPa), an indication graft biopsy should follow to securely diagnose graft fibrosis or other kinds of graft injury. LSM can be considered a pre-biopsy screening program to prioritize patients for Lbx. Personalized immunosuppression programs based on LSM screening and subsequent svLbx can help to improve the balance of necessary immunosuppression and immunosuppression associated side effects without relevant biopsy risk in OLT recipients (9). For example, knowledge of Ishak *F*2 fibrosis in a liver graft with normal liver values could lead to changes in immunosuppression, i.e., switching from high-dosage tacrolimus and MMF to low-dose tacrolimus and an mTOR inhibitor.

Although LSM is associated with inflammation and fibrosis before and after transplantation, we were not able to see an association of relevant liver graft injury in terms of 2016 BANFF criteria for the reduction of immunosuppression (BanffMini) and LSM. These findings match the recently published study by Vionnet et al. (8). However, the predictive fidelity for liver graft injury, defined by an elevated expression of rejection associated transcripts, could be increased by the combination of LSM with aminotransferase levels (8). Unfortunately, this combined prediction of LSM and aminotransferases could not be assessed in this cohort, because gene expression was not assessed in our clinical study.

Since all the performed Lbx were svLbx, an obvious limitation to this study was lack of sufficient availability of graft cirrhosis, so that safe cut-off values between various grades of graft fibrosis and cirrhosis could not be determined. Also, the patient number is at the lower limit of what is necessary to determine valid cut-off values for the identification of advanced fibrosis. Follow-up LSMs remain to be performed to evaluate changes in their results over time. Another important limitation was the small number of valid ARFI measurements for the left liver lobe, making it impossible to propose a specific cut-off value. ARFI measurements of the left lobe, however, are needed, since especially children regularly receive left-lobe split transplantations.

In conclusion, this study showed comparatively low cut-off values for the non-invasive detection of graft fibrosis in OLT recipients regardless of the underlying diseases in the absence of HCV reinfection. Transient elastography is primarily recommended due to lower rates of non-valid measurements and better correlation with graft fibrosis. In patients with LSM above 1.29 m/s and 7.5 kPa respectively, Lbx should be offered to determine the histopathological stage of fibrosis and evaluate changes in the immunosuppression regimen (9).

## Data Availability

The raw data supporting the conclusions of this article will be made available by the authors, without undue reservation.
