# Supplementary material for: Prospective comparison of liver stiffness measurement methods in surveillance biopsies after liver transplantation
Source: Front Transplant. 2023 Nov 17;2:1148195. doi: 10.3389/frtra.2023.1148195 (PMC11235307; doi:10.3389/frtra.2023.1148195)
Supplement: Supplementary file 1 [file Table1.docx]

**Supplemental Tables**

Supplemental Table 1: Baseline characteristics of training and validation cohorts

|  | ARFI training (n=39) | ARFI validation (n=30) | p-values ARFI | TE training (n=42) | TE validation (n=34) | p-values TE |
| --- | --- | --- | --- | --- | --- | --- |
| Age at biopsy (years) | 51 (18 – 72) | 58 (21 – 72) | 0.149 | 51 (20 – 72) | 53 (18 – 71) | 0.467 |
| Male gender n (%) | 19 (48.7) | 24 (80) | **0.008**^1^ | 23 (54.8) | 22 (64.7) | 0.380^1^ |
| BMI at biopsy (kg/m^2^) | 23.4 (18.6 – 31.3) | 24.1 (19.3 – 33.3) | 0.486 | 24.4 (18.4 – 34.4) | 25.2 (18.7 – 31.3) | 0.967 |
| Age at OLT (years) | 47 (4 – 67) | 49 (0 – 60) | 0.707 | 42 (0 – 67) | 47 (4 – 67) | 0.534 |
| Time from OLT to biopsy (months) | 94 (11 – 452) | 130 (11 – 406) | 0.090 | 71 (9 – 406) | 107 (10 – 306) | 0.839 |
| AST (U/l) | 24 (15 – 50) | 23 (12 – 55) | 0.694 | 26 (16 – 60) | 24 (8 – 51) | 0.191 |
| ALT (U/l) | 21 (13 – 47) | 21 (12 – 72) | 0.827 | 21 (10 – 72) | 21 (8 – 48) | 0.702 |
| AP (U/l) | 80 (32 – 473) | 85 (46 – 244) | 0.615 | 98 (46 – 263) | 82 (52 – 206) | 0.397 |
| GGT (U/l) | 22 (7 – 377) | 23 (11 – 180) | 0.961 | 24 (8 – 927) | 22 (7 – 220) | 0.504 |
| Bilirubin (µmol/l) | 9 (3 – 45) | 8 (3 – 55) | 0.334 | 9 (3 – 37) | 9 (3 – 55) | 0.805 |

Values are described as median (range), unless indicated differently.

p-values were calculated by Mann-Whitney-U test, except ^1^.

^1^ p-values calculated by Chi^2^ test.
